# Cloning and characterization of an adenoviral vector for highly efficient and doxycycline – suppressible expression of bioactive human single – chain interleukin 12 in colon cancer

**DOI:** 10.1186/1472-6750-7-35

**Published:** 2007-06-26

**Authors:** Holger Wulff, Thorsten Krieger, Karen Krüger, Ingrid Stahmer, Friedrich Thaiss, Hansjörg Schäfer, Andreas Block

**Affiliations:** 1 Department of Medicine, University of Hamburg, University Hospital Hamburg-Eppendorf, Martinistrasse 52, 20246 Hamburg, Germany; 2 Department of Immunology, University of Hamburg, University Hospital Hamburg-Eppendorf, Martinistrasse 52, 20246 Hamburg, Germany; 3 Institute of Pathology, University of Hamburg, University Hospital Hamburg-Eppendorf, Martinistrasse 52, 20246 Hamburg, Germany

## Abstract

**Background:**

Interleukin-12 (IL-12) is well characterized to induce cellular antitumoral immunity by activation of NK-cells and T-lymphocytes. However, systemic administration of recombinant human IL-12 resulted in severe toxicity without perceptible therapeutic benefit. Even though intratumoral expression of IL-12 leads to tumor regression and long-term survival in a variety of animal models, clinical trials have not yet shown a significant therapeutic benefit. One major obstacle in the treatment with IL-12 is to overcome the relatively low expression of the therapeutic gene without compromising the safety of such an approach. Our objective was to generate an adenoviral vector system enabling the regulated expression of very high levels of bioactive, human IL-12.

**Results:**

High gene expression was obtained utilizing the VP16 herpes simplex transactivator. Strong regulation of gene expression was realized by fusion of the VP16 to a tetracycline repressor with binding of the fusion protein to a flanking tetracycline operator and further enhanced by auto-regulated expression of its fusion gene within a bicistronic promoter construct. Infection of human colon cancer cells (HT29) at a multiplicity of infection (m.o.i.) of 10 resulted in the production of up to 8000 ng/10^6 ^cells in 48 h, thus exceeding any published vector system so far. Doxycycline concentrations as low as 30 ng/ml resulted in up to 5000-fold suppression, enabling significant reduction of gene expression in a possible clinical setting. Bioactivity of the human single-chain IL-12 was similar to purified human heterodimeric IL-12. Frozen sections of human colon cancer showed high expression of the coxsackie adenovirus receptor with significant production of human single chain IL-12 in colon cancer biopsies after infection with 3*10^7 ^p.f.u. Ad.3r-scIL12. Doxycycline mediated suppression of gene expression was up to 9000-fold in the infected colon cancer tissue.

**Conclusion:**

VP16 transactivator-mediated and doxycycline-regulated expression of the human interleukin-12 gene enables highly efficient and tightly controlled cytokine expression in human cancer. These data illustrate the potential of the described adenoviral vector system for the safe and superior expression of therapeutic genes in the treatment of colorectal cancer and other malignancies.

## Background

IL-12 has a major impact in the modulation of cellular immune response mechanisms. The heterodimeric cytokine enhances the proliferation and activation of natural killer cells (NK-cells) and the maturation of activated T lymphocytes to type 1 T helper cells (CD4^+^) and cytotoxic T lymphocytes (CD8^+^) [1-4] unveiling its potential for the treatment of infectious [5,6] and malignant diseases [7]. Induction of a cellular antitumoral immune response is directed not only against the primary lesion but also against distant organ metastases opening therapeutic options for those patients considered incurable at the present time. Tumor regression at the treatment site as well as in distant organ metastases was illustrated in 1997 utilizing a gene gun technique to incorporate plasmid DNA into intradermal tumors in mice [8]. In this particular tumor model IL-12 resulted in a superior antitumoral immune response and lasting systemic antitumoral immunity as compared with a variety of other cytokines (Interleukin-2, -4, -6, IFN-γ, TNF-α or GM-CSF). IL-12-mediated antitumoral immune response was also illustrated in a variety of different murine tumor models [9-14].

Systemic application of recombinant IL-12 resulted in a broad and undirected stimulation of the cellular immune system lacking antitumoral efficacy. Toxicity was mainly contributed by the induction of interferon-γ with anti-IFN-γ antibodies abolishing the lethal effect of systemic administration of recombinant IL-12 in mice [15]. Interferon-γ induction also contributes in part to the induction of a cellular antitumoral immune response since anti-IFN-γ antibodies diminish systemic antitumoral immunity [14]. Nevertheless, studies with IFN-γ receptor-knockout mice suggested an interferon-γ independent pathway of IL-12-mediated antitumoral immunity [16].

In humans the maximum tolerable dose (MTD) of IL-12 was determined to be 0.5 μg/kg body weight after intravenous administration [17,18] with reversion of advanced cancer associated defects in T-lymphocytes and NK cells [19]. Remarkably MTD was slightly lower after subcutaneous administration [20,21]. Repeated administration of IL-12 resulted in an increasing tolerance (priming effect) [20,22-24] which was in part due to increased STAT-4 (IFN-γ transcription activator) degradation.

In a phase II clinical trial repeated intravenous administration of recombinant IL-12 within less than 7 days resulted in leucopenia (65%), hyperbilirubinemia (47%), elevation of transaminases (47%), dyspnoa (29%) and lassitude (35%) with two fatalities due to hypovolemic shock caused by hemorrhagic colitis and sepsis [17].

In Hodgkin's lymphoma repeated intravenous or subcutaneous administration of recombinant IL-12 resulted in a 21% response rate with partial (PR) and complete regression (CR) [25]. Repeated local administration of rhIL12 in patients with T-cell lymphoma lead to an infiltration of the tumor tissue with inflammatory cells resulting in PR or CR in 50% of all cases [26]. IL-12 in patients with melanoma produced similar response rates [27]. However, results in renal cell and ovarian carcinoma were rather disappointing [28-31]. Colombo described in 1996 that the concentration of IL-12 at the tumor site might be crucial for the establishment of systemic antitumoral immunity [32].

With the development of vector systems, lasting as well as local expression of therapeutic genes have become feasible. Consequently, murine cancer cells either modified by IL-12 encoding plasmids or retrovirally transduced *ex vivo *[32-34] were able to immunize against the native cancer cell line and to protect against the tumor cells in consecutive challenge experiments. Even though direct intratumoral injection of IL-12 encoding plasmids resulted in significant immune response in different animal models [16,35,36], antitumoral response in a clinical trial remained marginal with no cure or long term immunity [37]. Poor transduction efficacy of plasmid vectors was overcome by the use of adenoviral vectors. Intratumoral injection of IL-12 encoding recombinant adenoviral vectors in mice resulted in a specific immunity against breast cancer and colon cancer [38,39]. Adoptive immunotransfer illustrated T-cell mediated, long term systemic immunity [40].

Gene therapeutic approaches utilizing the coexpression of both subunits for the generation of bioactive IL-12 were compromised by the formation of inhibitory p40 homodimers. This interference was prevented by the introduction of IL-12 fusion genes [34,41-43]. The potential of these constructs integrated in adenoviral expression systems for the treatment of gastrointestinal tumors was illustrated recently [44,45].

Considering the potential toxicity related to the expression of IL-12 and the necessity of high intratumoral concentrations, the scope of this study was to construct an adenoviral vector for highly efficient and regulated expression of a human single-chain IL-12. A modified E. coli operator system was utilized for tetracycline suppressible expression. High gene expression was enabled by the use of a VP16 herpes simplex virus transactivator fused to the E. coli Tet-repressor. Positioning of the trans-activating fusion protein was realized by Tet-operator upstream of the gene of interest [46]. This system was further enhanced by integration of a bidirectional promoter also expressing the transactivator in the absence of doxycycline [47]. In the presence of low concentrations of doxycycline the fusion protein immediately dissociates from the Tet-operator thru binding of doxycycline to the Tet-repressor domain with drastic reduction of VP16 mediated gene expression.

## Results

### Construction of suppressible vectors for the expression of human IL-12

**Figure 1:**
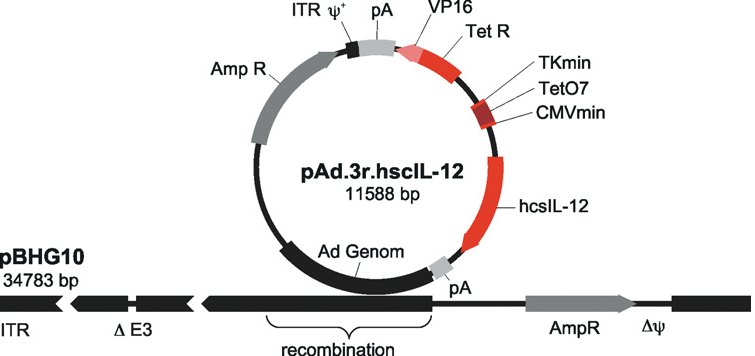
**Vector construction**. Construction of recombinant adenoviral vectors expressing human IL12 under control of doxycycline by calcium phosphate-mediated coprecipitation of the corresponding plasmids in 293 cells. VP16: Herpes simplex virus transactivator gene, TetR: Tetracycline repressor gene, TKmin: Thymidine kinase minimal promoter, TetO7: Tetracycline operator (seven copies), CMVmin: Cytomegalovirus minimal promoter, hscIL-12: human single-chain IL-12 gene, Amp R: ampicillin resistance gene, ITR: inverted terminal repeat, pA: poly-A from the bovine growth hormone gene.

The adenoviral expression plasmid pAd.3r-hscIL-12 was constructed as described, and corresponding recombinant E1/E3 deleted adenoviral vectors were generated by calcium phosphate mediated cotransfection with pBHG10 in 293-cells (figure 1). Plaques were purified and the adenovirus was amplified in 293-cells. The adenoviral titer was determined utilizing standard plaque assay procedures.

### Dox-dependent expression of human single-chain IL-12

**Figure 2:**
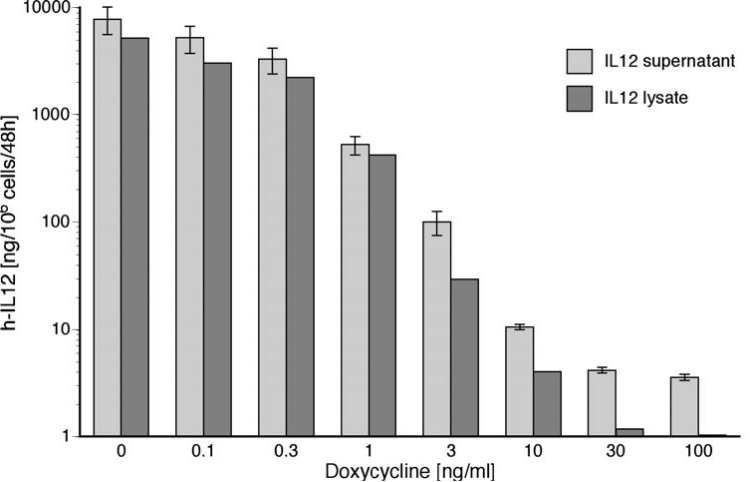
**Doxycycline dependent IL-12 expression**. Production of human interleukin-12 after infection of HT29 human colon cancer cells with Ad.3r-scIL12 at various concentrations of doxycycline.

We were able to illustrate the high adenoviral transduction efficacy in HT29 human colon cancer cells in previous publications [45,49,50]. These cells were infected with Ad.3r-hscIL12 at an m.o.i. of 10 followed by incubation for 48 hours at various concentrations of doxycycline. Human single-chain IL-12 was then determined in the cell lysate and the cell free culture supernatant. Substantial suppression was obtained at doxycycline concentrations as low as 3 ng/ml with maximum suppression levels of 2200-fold for IL-12 in the supernatant and 5000-fold in the cell lysate at 30 ng/ml doxycycline (figure 2). In the absence of dox, infection at an m.o.i. of 10 resulted in 2% IL-12 in relation to total soluble cellular protein (data not shown).

**Figure 3:**
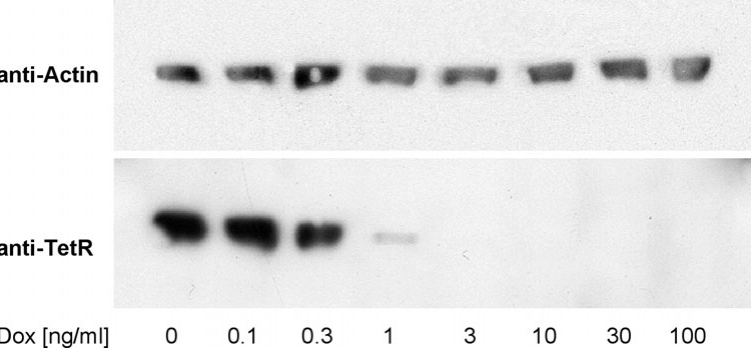
**Doxycycline dependent tTA expression**. Western blot analysis illustrates the positive feedback loop with dose dependent suppression of the tTA fusion protein in the presence of doxycycline.

Western blot analysis revealed significant suppression of TetR as detected with a TetR monoclonal antibody [46], illustrating down regulation of the transactivator proportional to the suppression of IL-12 expression in the presence of doxycycline (figure 3).

### Comparison of bioactivity

**Figure 4:**
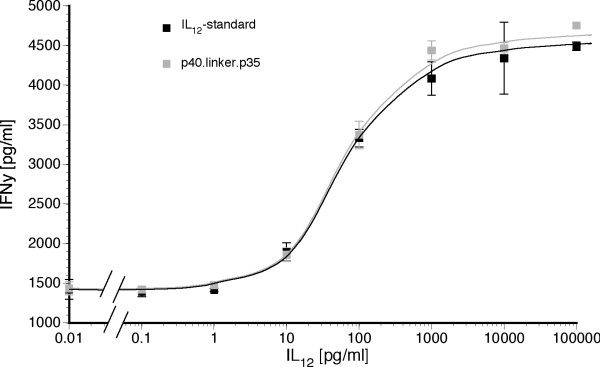
**Comparison of bioactivity**. Comparison of interferon-γ induction by incubation of mononuclear cells (MNC) with either adenovirally generated single-chain human interleukin-12 or purified standardized heterodimeric human interleukin-12 as quantified by ELISA.

Bioactivity of adenovirally-expressed human single-chain IL-12 was compared with purified heterodimeric p40/p35 standard in order to estimate the biological response in a clinical setting. Bioactivity was first evaluated by IFN-γ induction in human mononuclear cells after conditioning with CD3- and CD28- antibodies. Pretreated MNC were then incubated with hscIL-12 from the supernatant of adenovirally infected HT29 cells or with equivalent concentrations of a human heterodimeric IL-12 WHO-standard. Twenty-four hours after incubation at 37°C, expressed human interferon-γ was quantified by ELISA (figure 4). Bioactivity profiles did not reveal a significant difference. Interferon-γ induction was observed at IL-12 concentrations as low as 10 pg/ml with maximum induction at 1 ng/ml. ED_50 _was estimated to be 54 – 64 pg/ml IL-12 resulting in the induction of interferon-γ at 3000 pg/ml. Basal induction of interferon-γ in the absence of IL-12 was 1400 pg/ml, mainly due to prestimulation of MNC with CD3- and CD28-antibodies.

**Figure 5:**
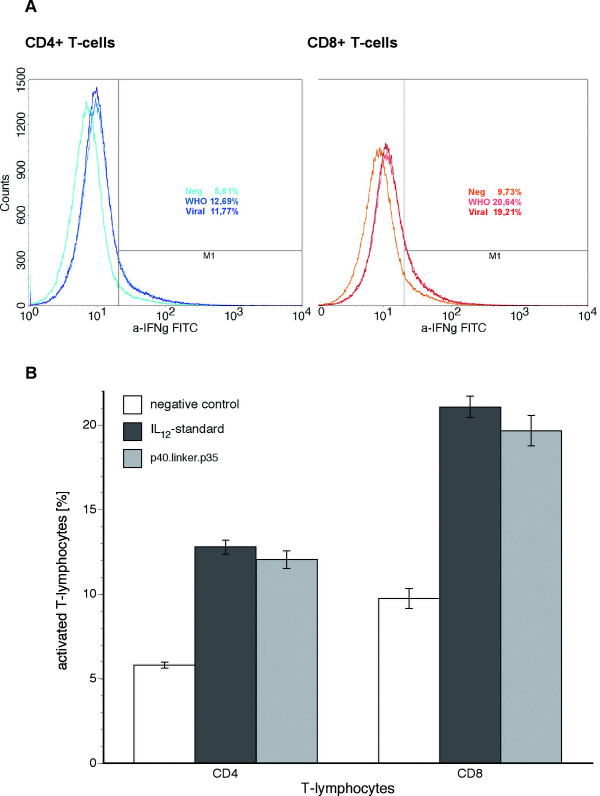
**FACS analysis**. FACS analysis of IFN-γ in CD3-gated MNC after preactivation by anti-CD3 and CD28 (negative control), additional stimulation with standardized heterodimeric human IL-12 (WHO IL12-standard) and adenovirally produced single-chain IL-12 (p40.linker.p35). A: comparison of representative samples, B: average of activated CD4+ and CD8+ T-lymphocytes (n = 3).

To further delineate whether the IFN-γ production was induced by IL-12, pre-stimulated MNC were incubated with a) native medium, b) heterodimeric IL-12, or c) viral IL-12 for 24 hours and brefeldin A for 20 hours to prevent the release of intracellular proteins including IFN-γ. The MNC were subsequently labeled by surface staining with fluorochrome-conjugated mAb's detecting CD3-APC/CD8 and CD69 followed by intracellular labeling with an anti-IFN-γ -FITC mAb and analyzed by flow cytometry. Recently activated T4- and T8-cells as defined by the expression of CD69 were analyzed for their IFN-γ producing profile. A representative IFN-γ expression analysis is shown in Figure 5A.

Basal production of IFN-γ was 5.81% ± 0.18% (CD4+) and 9.75% ± 0.58% (CD8+). Incubation with standardized heterodimeric human IL-12 resulted in 12.80% ± 0.42% (CD4+) and 21.08% ± 0.63% (CD8+) activation, incubation with adenovirally produced human single-chain IL-12 resulted in 12.05% ± 0.52% (CD4+) and 19.68% ± 0.90% (CD8+) production of IFN-γ. Both IL-12 preparations resulted in highly significant activation of CD4+ and CD8+ cells (p < 0.0001 each) with no significant difference in between heterodimeric IL-12 and single-chain IL-12 (CD4+: p = 0.124, CD8+: p = 0.092) concluding equal bioactivity for both cytokines (figure 5B).

### Coxsackie-adenovirus receptor expression and adenoviral transduction in human colon cancer

**Figure 6:**
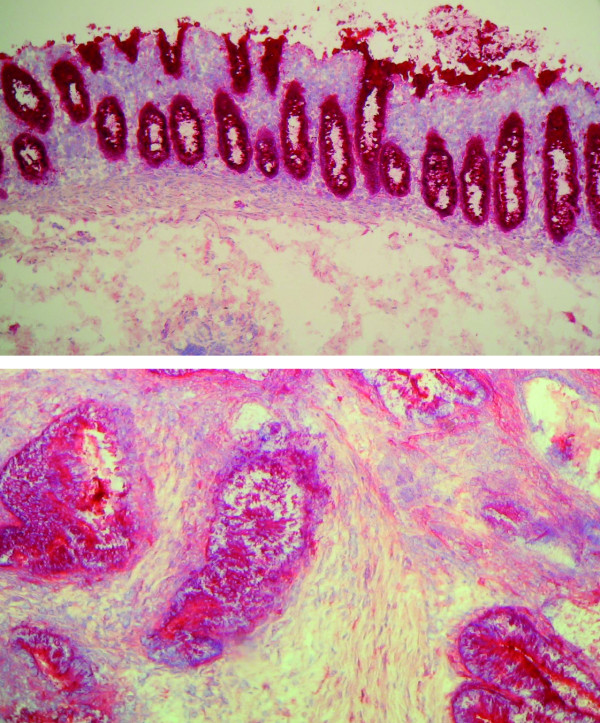
**CAR expression in human tissue**. Expression of the coxsackie adenovirus receptor (CAR) in biopsies of normal human colon (upper panel) and human colon cancer (lower panel). Red staining of CAR after reaction with a monoclonal anti-CAR antibody and an APAAP detection system.

**Figure 7:**
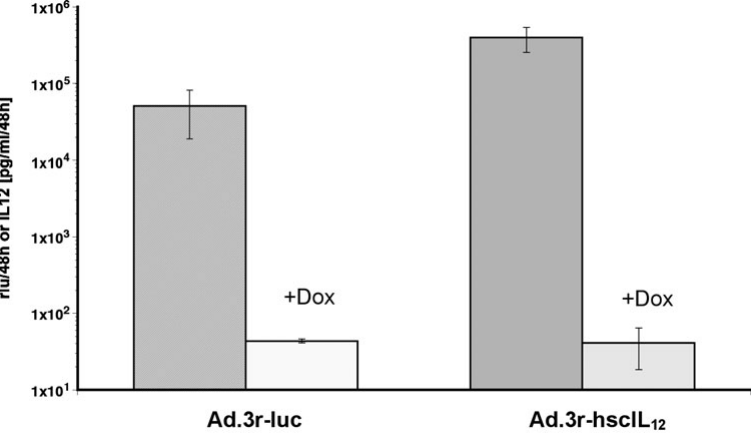
***Ex vivo *expression of luciferase and IL-12**. Luciferase and IL12 expression after *ex vivo *infection of human colon cancer biopsies with Ad.3r-luc or Ad.3r-hscIL12 in the presence (2 μg/ml) or absence of doxycycline.

Demonstrating high adenoviral transduction efficiency in a variety of human colon cancer cell lines [45], the question remained whether native human cancer tissue shows equal susceptibility towards adenoviral infection. Since adenoviral infection is mainly mediated by the Coxsackie-Adenovirus receptor (CAR), human colon and colon cancer tissues were stained for CAR expression. As illustrated in figure 6, normal colon epithelium as well as human colon cancer cells revealed high expression of the CAR using a monoclonal CAR-antibody and APAAP staining with no expression in the adjacent tissue. Staining applying a detection system based on horseradish peroxidase resulted in identical distribution (data not shown). Figure 7 illustrates transgene expression after infection of human colon cancer specimens with doxycycline suppressible adenoviral vectors. Luciferase and human IL-12 were significantly expressed in human colon cancer tissue in the absence of doxycycline. Addition of dox at a concentration of 2 μg/ml resulted in a 1200-fold suppression for non-secreted luciferase and 9700-fold suppression for secreted single-chain human IL-12.

## Discussion

Recent clinical trials utilizing recombinant IL-12 in the experimental treatment of human cancer illustrate the potential hazards of these approaches. Severe side effects such as hemorrhagic colitis and sepsis indicate the unacceptable risk associated with systemic and repeated administration of recombinant IL-12 in the treatment of cancer [17]. Local application is limited by the short half-life of the cytokine and showed some efficacy only in a sparse number of tumor entities. Therefore, local expression of an IL-12 gene is a promising approach to avoid such problems. Vector systems for the expression of human IL-12 did not result in significant clinical response yet, which is most likely due to weak transgene expression.

We were able to construct adenoviral vectors addressing these issues by incorporation of a highly potent and doxycycline-regulated transactivator for the expression of human single-chain IL-12. Infection of human colon cancer cells (HT29) at an m.o.i. of 10 resulted in the expression of 8000 ng/10^6 ^cells in 48 h. To our knowledge, these expression levels exceed any adenoviral vectors expressing human IL-12 as well as stably transduced cell lines published so far. Doxycycline concentrations as low as 30 ng/ml resulted in suppression levels of up to 5000-fold. With peak concentrations of 2.6 μg/ml 2 hours after oral application of 200 mg doxycycline and average serum levels of 1.11 μg/ml after 100 mg doxycycline daily [51], suitable concentrations of doxycycline are quickly obtainable for efficient and timely suppression of transgene expression. Addition of doxycycline to the drinking water completely abolished the otherwise lethal effect of these systemically administered murine IL-12 expressing vectors in mice (Block et al., Manuscript submitted).

Interferon-γ induction in human mononuclear cells after incubation with adenovirally expressed human single-chain IL-12 did not differ from induction with purified p35/p40 heterodimer at various concentrations. These results together with the comparative FACS analysis show similar bioactivity. Inhibition of bioactivity through the formation of p40 homodimers was avoided by expressing a IL-12 fusion protein rather than the separate subunits e.g. by utilizing an internal ribosome entry site [52,53]. Furthermore, the human single-chain IL-12 contains a secretory leader domain, resulting in efficient secretion independent of posttranslational processing and p35 subunit glycosylation [54,55].

Adenoviral transduction of human tissue requires the expression of the coxsackie adenovirus receptor (CAR) with vector internalization depending on alphavbeta3- and alphavbeta5-integrins. Expression of the CAR was shown in human colon epithelium as well as colon cancer illustrating the feasibility of adenoviral transduction and adenovirally mediated transgene expression. Adenoviral infection of freshly derived human colon cancer biopsies with 3*10^7 ^p.f.u. demonstrated efficient *ex vivo *transgene expression as well as highly significant doxycycline-mediated suppression in tumor tissue. Colon cancer differentiation dependent CAR expression and consecutive transgene expression remain to be determined and is currently under investigation.

These data further support the use of doxycycline-suppressible, highly efficient adenoviral vector systems for the treatment of human cancer. Vector related toxicity could probably be avoided by applying a significantly lower viral dose since transgene expression within these new promoter systems was shown to be more than 4000-fold higher as compared with the CMV-promoter commonly used in clinical trials. Tumor regression and systemic immunity after high intratumoral human IL-12 expression have to be addressed in a clinical setting. Even though oral administration of doxycycline can dramatically reduce IL-12 expression and related toxicity in mice, doxycycline mediated suppression of IL-12 expression in humans remains to be investigated. Serum half life of recombinant human IL-12 was determined to be 5 to 21 hours [18,20,23,56], thus possibly requiring additional precautions such as the temporary application of neutralizing anti IL-12 or interferon-γ antibodies in order to inactivate circulating cytokines.

## Conclusion

With expression levels exceeding the widely used viral CMV-promoter, combined with tight regulation of gene expression by low concentrations of doxycycline, we are confident that these adenoviral vectors will add to the feasibility and safety in the immunotherapy of colorectal cancer as well as other malignancies.

## Methods

### Cell lines

The 293-cell line was utilized for virus generation and amplification. 293-cells were maintained in HGDMEM (Gibco, Rockville, MD). HT29-cells were kept in McCoy 5A medium (Gibco). Human mononuclear blood cells (MNCs) were cultivated in RPMI1640 medium (Gibco). All cell culture media were supplemented with 10% fetal bovine serum (Gibco) or certified tetracycline free fetal bovine serum (Clontech, Palo Alto, CA), 1% L-glutamine and 1% penicillin/streptomycin (Gibco).

### Human colon cancer specimen

In accordance with national ethical guidelines issued by the German research foundation patients were informed about the study and gave their written consent at a minimum of 24 hours prior to surgery. Colon cancer specimens were removed according to standard surgical procedures. At least 8 colon cancer biopsies were taken from the vital area of each freshly prepared human colon cancer tissue utilizing a medium size endoscopic biopsy forceps. These samples were washed three times with PBS prior to *ex vivo *transduction with viral vectors.

### Construction of adenoviral expression plasmids

Preparation of plasmid DNA was performed utilizing a modified alkaline lysis protocol followed by purification through anion exchange column (Qiagen, Valencia, CA). Plasmid DNA was propagated in DH5a *E. coli*. Restriction endonucleases were obtained from Boehringer Mannheim (Ingelheim, Germany) and New England Biolabs (Ipswich, MA). The plasmid pSFG-hIL12.p40.linker-delta.p35 containing a human IL-12 fusion protein, was kindly provided by R.C. Mulligan (Children's hospital, Boston, MA) [42]. The backbone plasmid pAd.3r.pA was obtained by digestion of pAd.CMV.pA [45] with XbaI, blunting and SalI in order to remove the CMV promoter, the multiple cloning site, and the polyA. The previously described bicistronic promoter element was then released from pBIG3r after digestion with PvuII and SalI and ligated into the adenoviral expression plasmid. The cDNA encoding for human single-chain IL-12 (hscIL12) was released from pSFG-hIL12.p40.linker-delta.p35 through digestion with NcoI, refilling with T4 polymerase and subsequent digestion with NheI. pAd.3r.pA was digested with BamHI, refilled and digested with SpeI with ligation of the previously released hscIL12 encoding cDNA in between 3r and the poly A. The resulting adenoviral expression plasmid pAd.3r.hscIL-12 contains the cDNA encoding for a human IL-12 fusion protein under control of a bidirectional, doxycycline-suppressible promoter element, 5'-flanked by base pairs 1 to 456 and 3'-flanked by base pairs 3346 to 5864 of the Ad5 genome. Minimal TK-promoter-driven expression of the tTA was antiparallel and minimal CMV-promoter-driven expression of the human single-chain IL-12 gene was parallel to adenoviral E1 transcription. The complete expression cassette was sequenced and a base exchange repair performed at position 4059 (C/T) by PCR.

### Generation and amplification of the corresponding adenoviral vector

Recombinant, E1- and E3-deleted adenoviral vectors were generated by calcium phosphate mediated coprecipitation and homologous recombination of pAd.3r.hscIL12 with pBHG10 in 293-cells as described elsewhere [48]. Adenoviral vectors were propagated in 293-cells and purified by CsCl density gradient centrifugation. Titration of the purified virus was performed by plaque assay. The obtained titer for Ad.3r.hscIL12 was 3.6*10^9 ^p.f.u./ml. Viral DNA was isolated using the DNA Blood Mini Kit (Qiagen) and sequencing of the insert was done with an ABI 377 Sequenator (Perkin Elmer, Norwalk, CT) after asymmetrical amplification and labeling with BigDye Terminator Cycle Sequencing Mix (Applied Biosystems, Foster City, CA).

### Transfection and quantification of transgene expression

HT29 human colon cancer cells were seeded in six-well plates at a concentration of 10^6 ^cells/well 6 hours prior to infection. Tumor cells were infected in triplicates with purified recombinant adenovirus in 500 μl serum-free media for 1 hour followed by incubation with complete media and different doxycycline concentrations for another 48 hours. Human single-chain IL-12 was quantified in cell-free culture supernatant and cell lysate 48 hours after infection using a human IL-12 (P70) OptEIA ELISA (Pharmingen, San Jose, CA) assuming equal immunoreactivity as compared with the human p40/p35 heterodimer. Cells were lysed using 150 μl of culture lysis reagent (Promega, Madison, WI) according to the manufacturers protocol and IL-12 quantified as previously described.

Human colon cancer biopsies were infected with either Ad.3r-luc or Ad.hscIL12 for 4 hours followed by incubation in HGDMEM supplemented with certified tetracycline free fetal bovine serum (Clontech, Palo Alto, CA), 1% L-glutamine and 1% penicillin/streptomycin (Gibco) in the presence or absence of Doxycycline (2 μg/ml) for 48 hours. IL-12 was determined in the supernatant as described.

Luciferase expression was quantified by measuring luciferase activity in 20 μl of cell culture supernatant with a Bertold LB9507 luminometer and luciferase assay substrate (Promega). Standard curves were generated using recombinant firefly luciferase (Promega) diluted with CCLR to concentrations from 1 pg/ml to 300 ng/ml and a two-phase exponential association curve fitting was performed due to saturation at higher concentrations using the Prism software package (GraphPad Software, Inc, San Diego, CA).

### SDS-PAGE and immunoblotting

Cell lysates were resuspended in laemmli loading buffer and separated using gel electrophoresis (Nu PAGE Novex Bis-Tris Gel, Invitrogen). After blotting on Hybound-P PVDF membranes (Amersham, Buckinghamshire, UK), proteins were labeled with anti-β-actin (Sigma, St. Louis, MI) and anti-TetR (Clontech) monoclonal antibodies. Blots were then incubated with a peroxidase-linked sheep anti-mouse secondary antibody (Amersham) and proteins visualized through chemiluminescence by an ECL Western blotting detection Kit (Amersham).

### Bioassay

In order to characterize the bioactivity of virally expressed human single-chain IL-12, human peripheral blood mononuclear cells (MNCs) were purified from whole blood after written consent and in accordance with national ethical guidelines, following the LeucoSep-procedure (Greiner, Frickenhausen, Germany). Cell culture bottles were coated with anti-human CD3 antibodies (10 μg/ml in PBS, #MAB100, R&D Systems, Minneapolis, MN) and separated MNC were then incubated in the presence of an anti-human CD28 antibody (#AF-342-PB, R&D Systems) at 0.5 μg/ml for three days. Prestimulated MNC were seeded into 96 well plates at 4 × 10^4 ^cells/well and incubated in 125 μl with various concentrations of IL-12 either from adenovirally infected HT29 cell culture supernatant or WHO reference standard (#95/544, NIBSC, Herfordshire, UK). 24 hours later human interferon-γ was quantified in cell free supernatant using a human IFN-γ OptEIA ELISA (Pharmingen).

### FACS-analysis

CD3- and CD28-prestimulated MNC were seeded into 6 well plates at 10^6 ^cells/well and incubated with different IL-12 preparations at 100 pg/ml for 24 hours. 4 hours after the addition of IL-12 the golgi apparatus was blocked by brefeldin A (BFA) to prevent the export of newly synthesized INF-γ. Cells were then labeled utilizing the Fastimmune Intracellular Cytokine Detection Kit (#346049, Becton-Dickinson Biosciences, San Jose, CA). Briefly, cells were washed in PBS containing 0.5% fetal bovine serum and 0.1% NaN_3 _and resuspended in 0.5 ml FACS-Perm prior to incubation at room temperature for 10 minutes. Permeable cells were then stained with 20 μl of a DB Fastimmune anticytokine antibody solution (Anti-Hu-IFN-γ FITC/CD69PE/CD8PerCP-Cy5.5/CD3APC) for 30 minutes, washed and fixated in PFA (1% paraformaldehyde in PBS). Intracellular cytokine expression was performed by FACScalibur flow cytometry (BD Biosciences) and analyzed by cellquest software.

### Immunohistochemistry

Frozen sections of normal colon tissue and colon cancer tissue with a thickness between 5 and 10 microns were dried on coated slides at room temperature over night. After fixation with 4% paraformaldehyde for 10 minutes and incubation with 1% FCS for 30 minutes at room temperature, sample sections were then incubated with the mouse IgG_1 _monoclonal antibody Anti-CAR (clone RmcB, Upstate, NY) at 1 μg/ml in TBS supplemented with 1% fetal bovine serum for 1 hour at room temperature. Samples were incubated for 30 minutes at room temperature with rabbit anti-mouse IgG at 5 μg/ml. Immunostaining according to the alkaline phosphatase/anti-alkaline phosphatase (APAAP) protocol was done by incubating the sections with a mouse monoclonal APAAP complex (Dako, dilution 1:50) for 30 minutes at room temperature. The enzyme reaction was then detected by new fuchsin (Merck, Darmstadt, Germany), naphthol AS-BI (Sigma) and levamisol (Sigma) and sections were finally counterstained with hematoxylin.

## Authors' contributions

HW carried out the construction and characterization of human IL-12 expressing adenoviral vectors, TK and IS participated in the FACS analysis. KK carried out the *ex vivo *studies infecting human cancer tissue and quantifying gene expression, FT participated in the SDS page and immunoblotting, HS carried out the histochemistry and helped drafting the manuscript. AB conceived of the study, participated in its design and coordination and drafted the manuscript. All authors read and approved the final manuscript.

## References

[B1] Kobayashi M, Fitz L, Ryan M, Hewick RM, Clark SC, Chan S, Loudon R, Sherman F, Perussia B, Trinchieri G (1989). Identification and purification of natural killer cell stimulatory factor (NKSF), a cytokine with multiple biologic effects on human lymphocytes. J Exp Med.

[B2] Mehrotra PT, Wu D, Crim JA, Mostowski HS, Siegel JP (1993). Effects of IL-12 on the generation of cytotoxic activity in human CD8+ T lymphocytes. J Immunol.

[B3] Gately MK, Wolitzky AG, Quinn PM, Chizzonite R (1992). Regulation of human cytolytic lymphocyte responses by interleukin-12. Cell Immunol.

[B4] Trinchieri G (1993). Interleukin-12 and its role in the generation of TH1 cells. Immunol Today.

[B5] Kawakami K (2003). Promising immunotherapies with Th1-related cytokines against infectious diseases. J Infect Chemother.

[B6] Moreno SE, Alves-Filho JC, Alfaya TM, da Silva JS, Ferreira SH, Liew FY (2006). IL-12, but not IL-18, is critical to neutrophil activation and resistance to polymicrobial sepsis induced by cecal ligation and puncture. J Immunol.

[B7] Kilinc MO, Aulakh KS, Nair RE, Jones SA, Alard P, Kosiewicz MM, Egilmez NK (2006). Reversing tumor immune suppression with intratumoral IL-12: activation of tumor-associated T effector/memory cells, induction of T suppressor apoptosis, and infiltration of CD8+ T effectors. J Immunol.

[B8] Rakhmilevich AL, Janssen K, Turner J, Culp J, Yang NS (1997). Cytokine gene therapy of cancer using gene gun technology: superior antitumor activity of interleukin-12. Hum Gene Ther.

[B9] Brunda MJ, Luistro L, Warrier RR, Wright RB, Hubbard BR, Murphy M, Wolf SF, Gately MK (1993). Antitumor and antimetastatic activity of interleukin 12 against murine tumors. J Exp Med.

[B10] Nastala CL, Edington HD, McKinney TG, Tahara H, Nalesnik MA, Brunda MJ, Gately MK, Wolf SF, Schreiber RD, Storkus WJ (1994). Recombinant IL-12 administration induces tumor regression in association with IFN-gamma production. J Immunol.

[B11] Zou JP, Yamamoto N, Fujii T, Takenaka H, Kobayashi M, Herrmann SH, Wolf SF, Fujiwara H, Hamaoka T (1995). Systemic administration of rIL-12 induces complete tumor regression and protective immunity: response is correlated with a striking reversal of suppressed IFN-gamma production by anti-tumor T cells. Int Immunol.

[B12] Noguchi Y, Richards EC, Chen YT, Old LJ (1995). Influence of interleukin 12 on p53 peptide vaccination against established Meth A sarcoma. Proc Natl Acad Sci U S A.

[B13] Brunda MJ (1995). Role of IL12 as an anti-tumour agent: current status and future directions. Res Immunol.

[B14] Fujiwara H, Hamaoka T (1996). Antitumor and antimetastatic effects of interleukin 12. Cancer Chemother Pharmacol.

[B15] Ozmen L, Pericin M, Hakimi J, Chizzonite RA, Wysocka M, Trinchieri G, Gately M, Garotta G (1994). Interleukin 12, interferon gamma, and tumor necrosis factor alpha are the key cytokines of the generalized Shwartzman reaction. J Exp Med.

[B16] Schultz J, Pavlovic J, Strack B, Nawrath M, Moelling K (1999). Long-lasting anti-metastatic efficiency of interleukin 12-encoding plasmid DNA. Hum Gene Ther.

[B17] Leonard JP, Sherman ML, Fisher GL, Buchanan LJ, Larsen G, Atkins MB, Sosman JA, Dutcher JP, Vogelzang NJ, Ryan JL (1997). Effects of single-dose interleukin-12 exposure on interleukin-12-associated toxicity and interferon-gamma production. Blood.

[B18] Atkins MB, Robertson MJ, Gordon M, Lotze MT, DeCoste M, DuBois JS, Ritz J, Sandler AB, Edington HD, Garzone PD, Mier JW, Canning CM, Battiato L, Tahara H, Sherman ML (1997). Phase I evaluation of intravenous recombinant human interleukin 12 in patients with advanced malignancies. Clin Cancer Res.

[B19] Robertson MJ, Cameron C, Atkins MB, Gordon MS, Lotze MT, Sherman ML, Ritz J (1999). Immunological effects of interleukin 12 administered by bolus intravenous injection to patients with cancer. Clin Cancer Res.

[B20] Portielje JE, Kruit WH, Schuler M, Beck J, Lamers CH, Stoter G, Huber C, de Boer-Dennert M, Rakhit A, Bolhuis RL, Aulitzky WE (1999). Phase I study of subcutaneously administered recombinant human interleukin 12 in patients with advanced renal cell cancer. Clin Cancer Res.

[B21] Ohno R, Yamaguchi Y, Toge T, Kinouchi T, Kotake T, Shibata M, Kiyohara Y, Ikeda S, Fukui I, Gohchi A, Sugiyama Y, Saji S, Hazama S, Oka M, Ohnishi K, Ohhashi Y, Tsukagoshi S, Taguchi T (2000). A dose-escalation and pharmacokinetic study of subcutaneously administered recombinant human interleukin 12 and its biological effects in Japanese patients with advanced malignancies. Clin Cancer Res.

[B22] Sacco S, Heremans H, Echtenacher B, Buurman WA, Amraoui Z, Goldman M, Ghezzi P (1997). Protective effect of a single interleukin-12 (IL-12) predose against the toxicity of subsequent chronic IL-12 in mice: role of cytokines and glucocorticoids. Blood.

[B23] Motzer RJ, Rakhit A, Schwartz LH, Olencki T, Malone TM, Sandstrom K, Nadeau R, Parmar H, Bukowski R (1998). Phase I trial of subcutaneous recombinant human interleukin-12 in patients with advanced renal cell carcinoma. Clin Cancer Res.

[B24] Rakhit A, Yeon MM, Ferrante J, Fettner S, Nadeau R, Motzer R, Bukowski R, Carvajal DM, Wilkinson VL, Presky DH, Magram J, Gately MK (1999). Down-regulation of the pharmacokinetic-pharmacodynamic response to interleukin-12 during long-term administration to patients with renal cell carcinoma and evaluation of the mechanism of this "adaptive response" in mice. Clin Pharmacol Ther.

[B25] Younes A, Pro B, Robertson MJ, Flinn IW, Romaguera JE, Hagemeister F, Dang NH, Fiumara P, Loyer EM, Cabanillas FF, McLaughlin PW, Rodriguez MA, Samaniego F (2004). Phase II clinical trial of interleukin-12 in patients with relapsed and refractory non-Hodgkin's lymphoma and Hodgkin's disease. Clin Cancer Res.

[B26] Rook AH, Wood GS, Yoo EK, Elenitsas R, Kao DM, Sherman ML, Witmer WK, Rockwell KA, Shane RB, Lessin SR, Vonderheid EC (1999). Interleukin-12 therapy of cutaneous T-cell lymphoma induces lesion regression and cytotoxic T-cell responses. Blood.

[B27] Mortarini R, Borri A, Tragni G, Bersani I, Vegetti C, Bajetta E, Pilotti S, Cerundolo V, Anichini A (2000). Peripheral burst of tumor-specific cytotoxic T lymphocytes and infiltration of metastatic lesions by memory CD8+ T cells in melanoma patients receiving interleukin 12. Cancer Res.

[B28] Gollob JA, Mier JW, Veenstra K, McDermott DF, Clancy D, Clancy M, Atkins MB (2000). Phase I trial of twice-weekly intravenous interleukin 12 in patients with metastatic renal cell cancer or malignant melanoma: ability to maintain IFN-gamma induction is associated with clinical response. Clin Cancer Res.

[B29] Motzer RJ, Rakhit A, Thompson JA, Nemunaitis J, Murphy BA, Ellerhorst J, Schwartz LH, Berg WJ, Bukowski RM (2001). Randomized multicenter phase II trial of subcutaneous recombinant human interleukin-12 versus interferon-alpha 2a for patients with advanced renal cell carcinoma. J Interferon Cytokine Res.

[B30] Hurteau JA, Blessing JA, DeCesare SL, Creasman WT (2001). Evaluation of recombinant human interleukin-12 in patients with recurrent or refractory ovarian cancer: a gynecologic oncology group study. Gynecol Oncol.

[B31] Portielje JE, Lamers CH, Kruit WH, Sparreboom A, Bolhuis RL, Stoter G, Huber C, Gratama JW (2003). Repeated administrations of interleukin (IL)-12 are associated with persistently elevated plasma levels of IL-10 and declining IFN-gamma, tumor necrosis factor-alpha, IL-6, and IL-8 responses. Clin Cancer Res.

[B32] Colombo MP, Vagliani M, Spreafico F, Parenza M, Chiodoni C, Melani C, Stoppacciaro A (1996). Amount of interleukin 12 available at the tumor site is critical for tumor regression. Cancer Res.

[B33] Tamura T, Nishi T, Goto T, Takeshima H, Dev SB, Ushio Y, Sakata T (2001). Intratumoral delivery of interleukin 12 expression plasmids with in vivo electroporation is effective for colon and renal cancer. Hum Gene Ther.

[B34] Lode HN, Dreier T, Xiang R, Varki NM, Kang AS, Reisfeld RA (1998). Gene therapy with a single chain interleukin 12 fusion protein induces T cell-dependent protective immunity in a syngeneic model of murine neuroblastoma. Proc Natl Acad Sci U S A.

[B35] Heinzerling LM, Feige K, Rieder S, Akens MK, Dummer R, Stranzinger G, Moelling K (2001). Tumor regression induced by intratumoral injection of DNA coding for human interleukin 12 into melanoma metastases in gray horses. J Mol Med.

[B36] Heinzerling L, Dummer R, Pavlovic J, Schultz J, Burg G, Moelling K (2002). Tumor regression of human and murine melanoma after intratumoral injection of IL-12-encoding plasmid DNA in mice. Exp Dermatol.

[B37] Heinzerling L, Burg G, Dummer R, Maier T, Oberholzer PA, Schultz J, Elzaouk L, Pavlovic J, Moelling K (2005). Intratumoral injection of DNA encoding human interleukin 12 into patients with metastatic melanoma: clinical efficacy. Hum Gene Ther.

[B38] Caruso M, Pham-Nguyen K, Kwong YL, Xu B, Kosai KI, Finegold M, Woo SL, Chen SH (1996). Adenovirus-mediated interleukin-12 gene therapy for metastatic colon carcinoma. Proc Natl Acad Sci U S A.

[B39] Bramson JL, Hitt M, Addison CL, Muller WJ, Gauldie J, Graham FL (1996). Direct intratumoral injection of an adenovirus expressing interleukin-12 induces regression and long-lasting immunity that is associated with highly localized expression of interleukin-12. Hum Gene Ther.

[B40] Mazzolini G, Qian C, Narvaiza I, Barajas M, Borras-Cuesta F, Xie X, Duarte M, Melero I, Prieto J (2000). Adenoviral gene transfer of interleukin 12 into tumors synergizes with adoptive T cell therapy both at the induction and effector level. Hum Gene Ther.

[B41] Lee YL, Tao MH, Chow YH, Chiang BL (1998). Construction of vectors expressing bioactive heterodimeric and single-chain murine interleukin-12 for gene therapy. Hum Gene Ther.

[B42] Lieschke GJ, Rao PK, Gately MK, Mulligan RC (1997). Bioactive murine and human interleukin-12 fusion proteins which retain antitumor activity in vivo. Nat Biotechnol.

[B43] Anderson R, Macdonald I, Corbett T, Hacking G, Lowdell MW, Prentice HG (1997). Construction and biological characterization of an interleukin-12 fusion protein (Flexi-12): delivery to acute myeloid leukemic blasts using adeno-associated virus. Hum Gene Ther.

[B44] Waehler R, Ittrich H, Mueller L, Krupski G, Ameis D, Schnieders F (2005). Low-dose adenoviral immunotherapy of rat hepatocellular carcinoma using single-chain interleukin-12. Hum Gene Ther.

[B45] Block A, Puls F, Muller J, Milasinovic D, Igelmann D, Schafer P, Kupfermann N, Schmoldt A, Ameis D, Greten H (2003). Highly suppressible expression of single-chain interleukin-12 by doxycycline following adenoviral infection with a single-vector Tet-regulatory system. J Gene Med.

[B46] Gossen M, Bujard H (1992). Tight control of gene expression in mammalian cells by tetracycline-responsive promoters. Proc Natl Acad Sci U S A.

[B47] Baron U, Freundlieb S, Gossen M, Bujard H (1995). Co-regulation of two gene activities by tetracycline via a bidirectional promoter. Nucleic Acids Res.

[B48] Block A, Freund CT, Chen SH, Nguyen KP, Finegold M, Windler E, Woo SL (2000). Gene therapy of metastatic colon carcinoma: regression of multiple hepatic metastases by adenoviral expression of bacterial cytosine deaminase. Cancer Gene Ther.

[B49] Block A, Milasinovic D, Mueller J, Schaefer P, Schaefer H, Greten H (2002). Amplified Muc1-specific gene expression in colon cancer cells utilizing a binary system in adenoviral vectors. Anticancer Res.

[B50] Lavda M, Clausnitzer CE, Walters JD (2004). Distribution of systemic ciprofloxacin and doxycycline to gingiva and gingival crevicular fluid. J Periodontol.

[B51] Ling P, Gately MK, Gubler U, Stern AS, Lin P, Hollfelder K, Su C, Pan YC, Hakimi J (1995). Human IL-12 p40 homodimer binds to the IL-12 receptor but does not mediate biologic activity. J Immunol.

[B52] Heinzel FP, Hujer AM, Ahmed FN, Rerko RM (1997). In vivo production and function of IL-12 p40 homodimers. J Immunol.

[B53] Murphy FJ, Hayes MP, Burd PR (2000). Disparate intracellular processing of human IL-12 preprotein subunits: atypical processing of the P35 signal peptide. J Immunol.

[B54] Carra G, Gerosa F, Trinchieri G (2000). Biosynthesis and posttranslational regulation of human IL-12. J Immunol.

[B55] Bajetta E, Del Vecchio M, Mortarini R, Nadeau R, Rakhit A, Rimassa L, Fowst C, Borri A, Anichini A, Parmiani G (1998). Pilot study of subcutaneous recombinant human interleukin 12 in metastatic melanoma. Clin Cancer Res.

[B56] Graham FL, van der Eb AJ (1973). Transformation of rat cells by DNA of human adenovirus 5. Virology.

